# Ameliorating Fibrosis in Murine and Human Tissues with END55, an Endostatin-Derived Fusion Protein Made in Plants

**DOI:** 10.3390/biomedicines10112861

**Published:** 2022-11-09

**Authors:** Logan Mlakar, Sara M. Garrett, Tomoya Watanabe, Matthew Sanderson, Tetsuya Nishimoto, Jonathan Heywood, Kristi L. Helke, Joseph M. Pilewski, Erica L. Herzog, Carol Feghali-Bostwick

**Affiliations:** 1 Division of Rheumatology, Department of Medicine, Medical University of South Carolina, Charleston, SC 29425, USA; 2 Department of Comparative Medicine, Medical University of South Carolina, Charleston, SC 29425, USA; 3 Division of Pulmonary, Allergy and Critical Care Medicine, Department of Medicine, University of Pittsburgh, Pittsburgh, PA 15261, USA; 4 Yale ILD Center of Excellence, Department of Medicine, Yale School of Medicine, New Haven, CT 06519, USA

**Keywords:** endostatin, peptides, bleomycin, fibrosis, idiopathic pulmonary fibrosis, systemic sclerosis, extracellular matrix

## Abstract

Organ fibrosis, particularly of the lungs, causes significant morbidity and mortality. Effective treatments are needed to reduce the health burden. A fragment of the carboxyl-terminal end of collagen XVIII/endostatin reduces skin and lung fibrosis. This fragment was modified to facilitate its production in plants, which resulted in the recombinant fusion protein, END55. We found that expression of END55 had significant anti-fibrotic effects on the treatment and prevention of skin and lung fibrosis in a bleomycin mouse model. We validated these effects in a second mouse model of pulmonary fibrosis involving inducible, lung-targeted expression of transforming growth factor β1. END55 also exerted anti-fibrotic effects in human lung and skin tissues maintained in organ culture in which fibrosis was experimentally induced. The anti-fibrotic effect of END55 was mediated by a decrease in the expression of extracellular matrix genes and an increase in the levels of matrix-degrading enzymes. Finally, END55 reduced fibrosis in the lungs of patients with systemic sclerosis (SSc) and idiopathic pulmonary fibrosis (IPF) who underwent lung transplantation due to the severity of their lung disease, displaying efficacy in human tissues directly relevant to human disease. These findings demonstrate that END55 is an effective anti-fibrotic therapy in different organs.

## 1. Introduction

Chronic fibrosing lung and skin diseases, such as idiopathic pulmonary fibrosis (IPF) and systemic sclerosis (SSc; scleroderma), affect millions of people and account for nearly 45% of deaths in the industrialized world [1,2,3]. IPF typically affects older men, resulting in progressive declines in lung function that reduce life expectancy. Conversely, SSc predominantly affects women, with primary deaths involving compromised pulmonary involvement [4,5,6]. Despite these divergent clinical phenotypes, convergent fibrotic mechanisms may be involved. Therefore, we need to identify therapies with efficacy in both IPF and SSc.

A hallmark of fibrosis shared by different organs is the excess accumulation of extracellular matrix (ECM) proteins, such as collagen types I and III and fibronectin. This excess accumulation alters the normal architecture of the organ, often impairing function and contributing to morbidity and mortality [7,8]. Secretion of ECM proteins is mediated, in part, by fibroblasts differentiating into myofibroblasts. This differentiation can be stimulated by exogenous factors, such as transforming growth factor β1 (TGFβ1) [9]. However, interventions targeting this pathway have had mixed results clinically [10,11]. The U.S. Food and Drug Administration (FDA) has approved two treatments for pulmonary fibrosis, including pirfenidone for IPF [12] and nintedanib for IPF [13,14] and SSc [15]). These treatments may slow disease progression, but neither halts the progression nor reverses established disease. Therefore, we critically need to develop more effective therapies for these and related diseases.

Plant production of recombinant biotherapeutic proteins has emerged as a rapid, efficient, and cost-effective strategy. Plants are used to efficiently produce vaccine antigens, enzymes, monoclonal antibodies, and biologically active hormones for scientific research and clinical products. Plant-based recombinant expression methods for creating and screening libraries of proteins to optimize functional properties have several advantages [16,17,18]. These advantages include low cost, safe profiles, proper protein folding and posttranslational modifications, and the absence of potential contaminating animal pathogens. In 2012, the FDA-approved the first plant-manufactured therapeutic protein for human use [18]; however, this powerful technology has not yet been used to develop antifibrotic therapies.

We and others have described the anti-fibrotic effects of full-length endostatin in vitro, ex vivo, and in vivo [19,20]. Endostatin is a 20-kDa fragment cleaved from the carboxyl-terminal domain of the non-collagenous NC1 domain of collagen XVIII, a multiplexin located in the perivascular space [21,22]. Endostatin specifically inhibits endothelial proliferation, exhibits anti-tumor activity, and has potent anti-angiogenic activity via its amino-terminal end [21,23], but not its carboxyl-terminal region [20]. We found that the carboxyl-terminal region (E3) abolishes TGFβ1-induced fibrosis in human skin explants and reduces skin and lung fibrosis in murine models of fibrosis [20].

In this study, a modified fusion protein, deemed END55, engineered for production in plants and with improved formulation properties, is assessed for anti-fibrotic capabilities in two mouse models and two human organ models of fibrosis.

## 2. Results

### 2.1. Measurement of END55 Targets to Develop a Potency Assay

The E3 region of endostatin reduces lung fibrosis induced by bleomycin and skin fibrosis induced by bleomycin and TGFβ1 in vivo [20,24]. A modified fusion protein homologous to this E3 region, END55, was engineered in order to improve manufacturing. END55 contains proprietary modifications, including a leader peptide, an internal amino acid substitution, and fusion to the Fc region of human IgG1. These modifications increase the solubility and stability of the molecule, confer protease resistance (to retain biological activity), and enhance its anti-fibrotic activity. To identify proteins regulated by END55 that could serve to compare the activity of different peptide preparations (i.e., cell-based potency assays), we measured several markers in fibroblasts and mouse serum. These markers included connective tissue growth factor (CTGF), matrix metalloproteinases 1 (MMP1), and lysyl oxidase (LOX).

**Figure 1:**
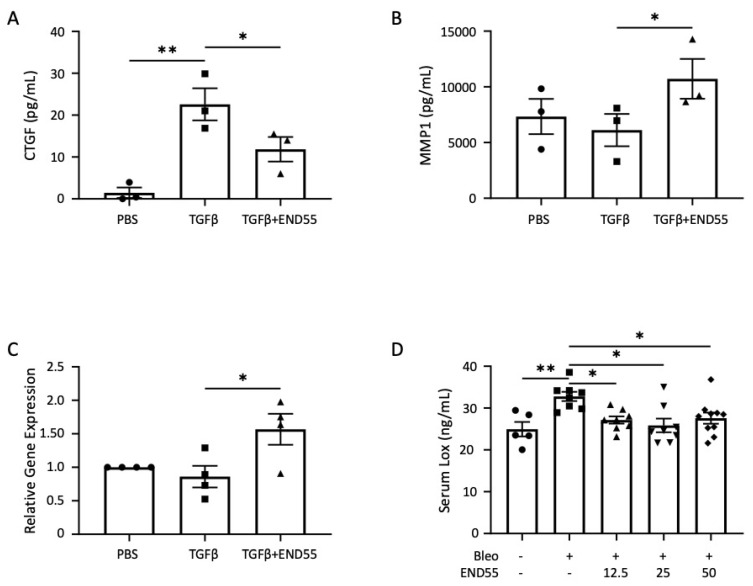
**Development of a potency assay for END55.** (**A**) Secreted CTGF protein from human fetal fibroblasts (MRC5) was measured by ELISA following 24 h culture with PBS (vehicle), TGFβ (5 ng/mL), or TGFβ+END55 (200 µg/mL). N = 3. (**B**) MMP1 protein measured in MRC5 supernatants after incubation with PBS (vehicle), TGFβ (5 ng/mL), or TGFβ+END55 (200 µg/mL) for 24 h. N = 3. (**C**) Relative gene expression of *MMP1* (normalized to *B2M*) in fibroblasts treated with PBS (vehicle), TGFβ (5 ng/mL), or TGFβ+END55 (100 µg/mL) for 1 h. N = 4. (**D**) Mouse LOX levels measured in 21-day serum by ELISA. N = 5–10. Data are represented as mean ± SD. * *p* < 0.05; ** *p* < 0.01. Bleo, bleomycin.

CTGF is a gene highly regulated by TGFβ, and it has been implicated in fibroblast activation and correlated with the severity of pulmonary fibrosis [4,11,25]. END55 significantly reduced TGFβ-stimulated secretion of the CTGF protein from human fibroblasts (Figure 1A).

Remodeling of the ECM architecture is mediated, in part, by proteases, including the prototypic collagenase MMP1 [26]. END55 rescued the TGFβ-driven decrease in secreted MMP1 (Figure 1B) and MMP1 gene expression (Figure 1C) in fibroblasts.

Crosslinking of matrix components, such as collagen and elastin, through the conversion of lysine moieties to aldehydes by the enzyme LOX, reduces matrix proteolysis in skin and lung fibrosis [27,28,29]. Circulating LOX levels are higher in patients with SSc and correlate with the severity and extent of skin involvement [29]. LOX levels are also higher in the lung tissues of SSc patients, and they can directly promote a fibrotic phenotype in vivo and ex vivo in human lung and skin tissues [30]. In a murine model of bleomycin lung fibrosis, serum LOX levels were used as a biomarker of response to therapy. Circulating LOX levels were significantly higher with bleomycin, which was reduced by treatment with END55 at multiple doses (Figure 1D). These results support our recent findings that LOX is a circulating biomarker of the response to the free peptide, E4, in the bleomycin-induced murine model of lung fibrosis [30]. These results show the feasibility of a cell-based assay to monitor the response to END55. The results also validate using circulating LOX levels to monitor the response to treatment with END55. Because END55 regulated select fibrotic markers from TGFβ- and bleomycin-mediated fibrosis, we further assessed its efficacy in two murine fibrosis models and two human tissue models.

### 2.2. END55 Ameliorates Bleomycin-Induced Lung Fibrosis

**Figure 2:**
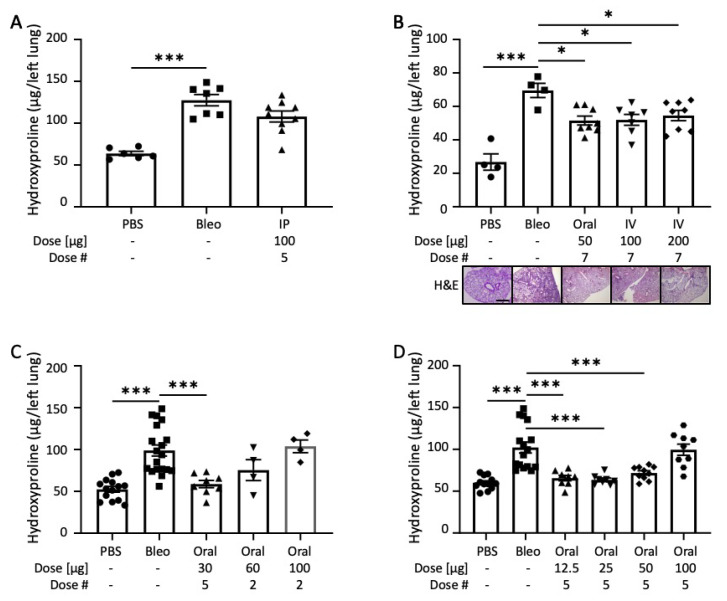
**END55 reduces bleomycin-induced pulmonary fibrosis.** (**A**) Oropharyngeal bleomycin was delivered on day zero along with END55 delivered by IP injection (100 µg every 4 days for 5 doses). Hydroxyproline was measured in left-lung tissues on day 21. N = 6–9. (**B**) Oropharyngeal bleomycin was delivered on day zero along with END55 delivered by oral gavage (50 µg) or IV injection (100 µg or 200 µg) every other day for 7 doses. Hydroxyproline was measured in left-lung tissues on day 14. Representative hematoxylin and eosin (H&E) staining is shown for comparison (scale bar, 300 µm; 2.5× magnification). N = 4–8. (**C**) Oropharyngeal bleomycin was delivered on day zero, along with END55 delivered by oral gavage in 5 doses (30 µg every 3 or 4 days) or 2 doses (either 60 or 100 µg on day 0 and day 5). Hydroxyproline was measured in left-lung tissues on day 21. N = 4–19. (**D**) Oropharyngeal bleomycin was delivered on day zero along with END55 delivered by oral gavage in 5 doses (12.5, 25, 50, and 100 µg). Hydroxyproline was measured in left-lung tissues on day 21. N = 8–16. Data are represented as mean ± SD. * *p* < 0.05, *** *p* < 0.001.

In order to assess the biological effectiveness of END55, we tested the fusion protein in a bleomycin lung fibrosis model in vivo. Mice receiving bleomycin via the oropharyngeal route had significantly more hydroxyproline content in their lungs after 21 days compared to mice treated with phosphate-buffered saline (PBS) (Figure 2A). Concurrent administration of bleomycin with intraperitoneal injections of END55 (five equally spaced doses of 100 µg each) yielded reduced hydroxyproline content and histological evidence of lung fibrosis, as assessed using Ashcroft scoring [31] (Supplementary Figure S1). Two additional routes of administration for END55 were also tested: oral gavage and intravenous (IV) injection. Bleomycin-treated mice were given END55 via oral gavage (50 µg) or IV injection (100 µg and 200 µg) every other day for seven treatments, starting on day zero. Lung tissues were harvested after 14 days (Figure 2B). Hydroxyproline content was significantly higher with bleomycin treatment after 14 days, as can also be seen histologically with representative hematoxylin and eosin staining of lung tissues. All three dosing schema of END55 significantly reduced hydroxyproline content of lung tissues (Figure 2B). Decreases in hydroxyproline levels were paralleled by significant decreases in the gene expression levels of *Col1a1*, *Col1a2*, and *FN1* in mouse lungs (Supplementary Figure S2).

We next tested the effect of END55 administered by oral gavage and less frequent dosing. Oral END55 at 30 µg every 3 to 4 days significantly reduced lung hydroxyproline levels induced by bleomycin (Figure 2C). Higher doses of END55 at less frequent treatment modalities (one dose along with bleomycin, followed by another dose after 5 days) modestly reduced lung hydroxyproline levels compared to bleomycin treatment alone.

In a dose-escalation study, we administered END55 via oral gavage every 4 days at doses between 12.5 µg and 100 µg along with bleomycin (Figure 2D). Lung tissues were harvested 21 days later. The three lowest concentrations of END55 (12.5 µg, 25 µg, and 50 µg) significantly reduced the effects of bleomycin on hydroxyproline content in the lungs of mice. Thus, END55 reduces the effects of bleomycin lung fibrosis in vivo via different timing, doses, and routes of administration.

### 2.3. END55 Reverses Bleomycin-Induced Lung Fibrosis

**Figure 3:**
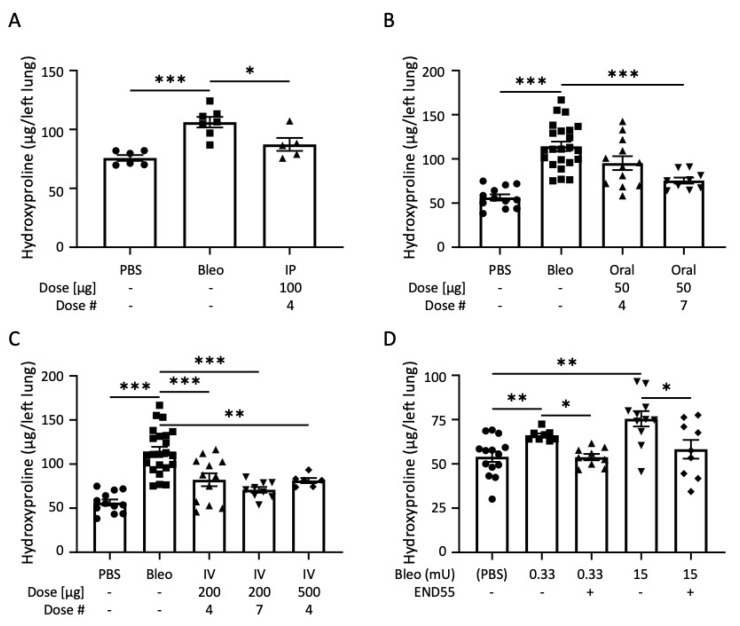
**END55 reverses bleomycin-induced pulmonary fibrosis.** (**A**) Four days after oropharyngeal bleomycin, END55 (100 µg) was delivered intraperitoneally and every 3 days for 4 doses. Hydroxyproline was measured in left-lung tissues on day 17. N = 5–7. (**B**) Bleomycin was delivered oropharyngeally on day zero, and END55 was delivered by oral gavage starting on day 7 (50 µg in 4 or 7 doses). Hydroxyproline was measured in left-lung tissues on day 21. N = 9–23. (**C**) Oropharyngeal bleomycin was delivered on day zero, and END55 was delivered by IV injection starting on day 7 (200 µg or 500 µg in 4 or 7 doses). Hydroxyproline was measured in left-lung tissues on day 21. N = 6–23. (**D**) Mice were implanted with dorsomedial pumps containing bleomycin (low-dose, 0.33 mU; high-dose, 15 mU) for 1 week, followed by END55 delivery via pump. Hydroxyproline was measured in left lung tissues treated with low-dose bleomycin on day 35 and with high-dose bleomycin on day 28. N = 8–14. Data are represented as mean ± SD. * *p* < 0.05, ** *p* < 0.01, *** *p* < 0.001.

Because we found that END55 prevented fibrosis, we examined its ability to reverse bleomycin-induced lung fibrosis in vivo. We tested the ability of various routes of END55 administration to ameliorate ongoing lung fibrosis after delivering bleomycin with an oropharyngeal dose or dorsomedial mini-osmotic pump (Figure 3). Beginning 4 days after administration of bleomycin, intraperitoneal (IP) injection of END55 (100 µg every 3 days for a total of four doses) significantly reduced hydroxyproline content in day 17 lung tissues in vivo (Figure 3A). END55 (50 µg), delivered by oral gavage starting 7 days after bleomycin, trended toward reduced hydroxyproline after four doses and significantly reduced hydroxyproline after seven doses in day 21 lung tissues (Figure 3B). END55 was also administered by IV in another group of mice. Three different IV dosing schema were analyzed: four doses of 200 µg, seven doses of 200 µg, and four doses of 500 µg END55 starting 7 days after bleomycin. IV END55 reduced hydroxyproline in the lungs of mice treated with all concentrations and doses (Figure 3C). These findings support the notion that END55 reverses ongoing lung fibrosis via different administration routes.

### 2.4. END55 Reverses Established Bleomycin Lung Fibrosis

In order to determine the extent to which END55 can reverse established fibrosis, we mimicked different levels of fibrosis by using mini-osmotic pumps implanted dorsomedially to deliver low-dose (0.33 mU) and high-dose (15 mU) bleomycin. Bleomycin was delivered via pump for 7 days, followed by pump-delivered vehicle or END55 (2.59 mg in 100 µL) for the next 7 days. Hydroxyproline content of lung tissues was examined at 35 days for the lower bleomycin dose and at 28 days for the higher bleomycin dose. Both low-dose and high-dose bleomycin increased the hydroxyproline content in lung tissues of mice (Figure 3D). Treatment with END55 significantly reduced hydroxyproline content in both low-dose and high-dose bleomycin groups, suggesting that END55 can reverse established mild and severe fibrosis.

### 2.5. END55 Reduces Skin Thickness in Mice with Established Fibrosis

**Figure 4:**
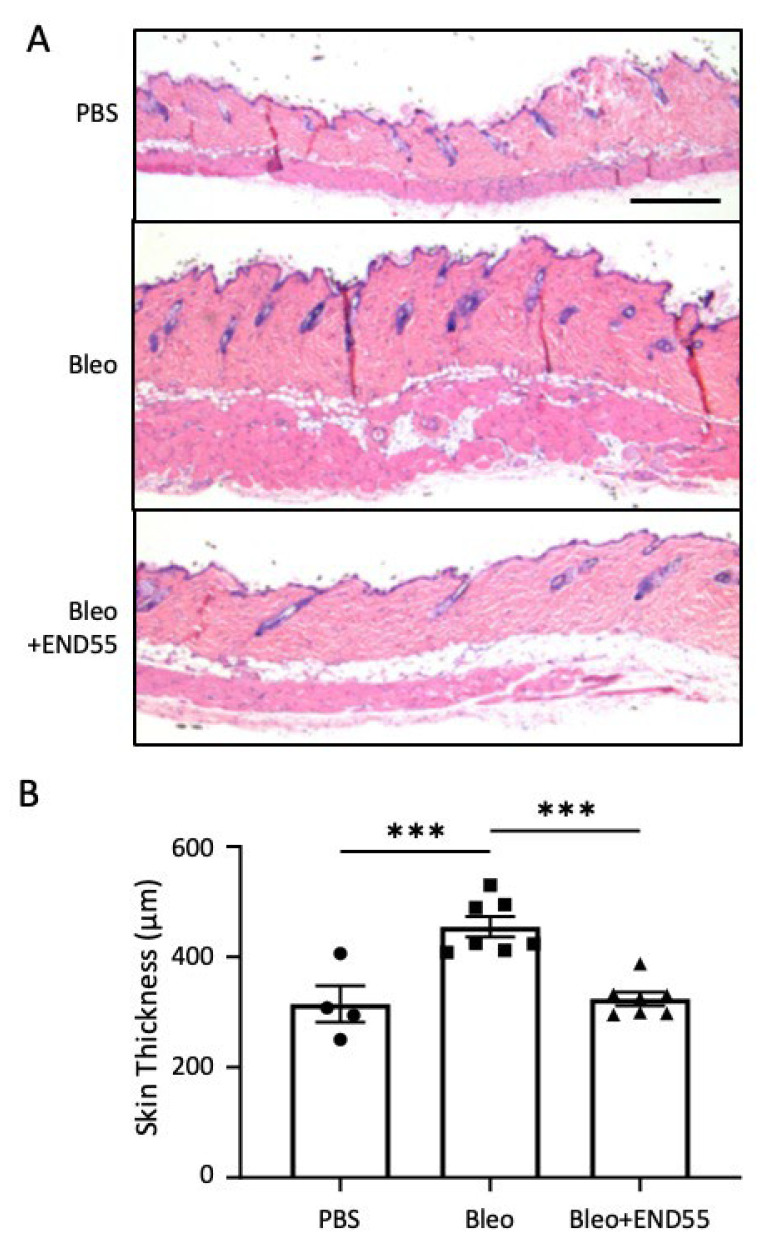
**END55 reduces skin thickness induced by bleomycin in mice.** (**A**) Representative hematoxylin and eosin staining (scale bar, 500 µm; 2.5× magnification) and quantification of thickness (**B**) of skin sections in day 35 tissues from mice treated with PBS (vehicle) or bleomycin for 7 days via dorsomedial pump, followed by treatment with PBS (vehicle) or END55 for 7 days via dorsomedial pump. N = 4–7. Data are represented as mean ± SD. *** *p* < 0.001.

As a secondary measure of fibrosis resolution by END55, we examined skin thickness pericentral to the implantation site in mice receiving bleomycin for 1 week followed by END55 for 1 week via dorsomedial pumps (Figure 4). Hematoxylin and eosin staining revealed that dermal skin was significantly thicker with bleomycin compared to vehicle (PBS). END55 administered for 1 week after bleomycin significantly reduced skin thickness, comparable to the dermal thickness of PBS-treated mice. These findings demonstrate the therapeutic efficacy of END55 and the complete resolution of fibrosis in another tissue.

### 2.6. END55 Prevents and Ameliorates TGFβ-Induced Fibrosis In Vivo

**Figure 5:**
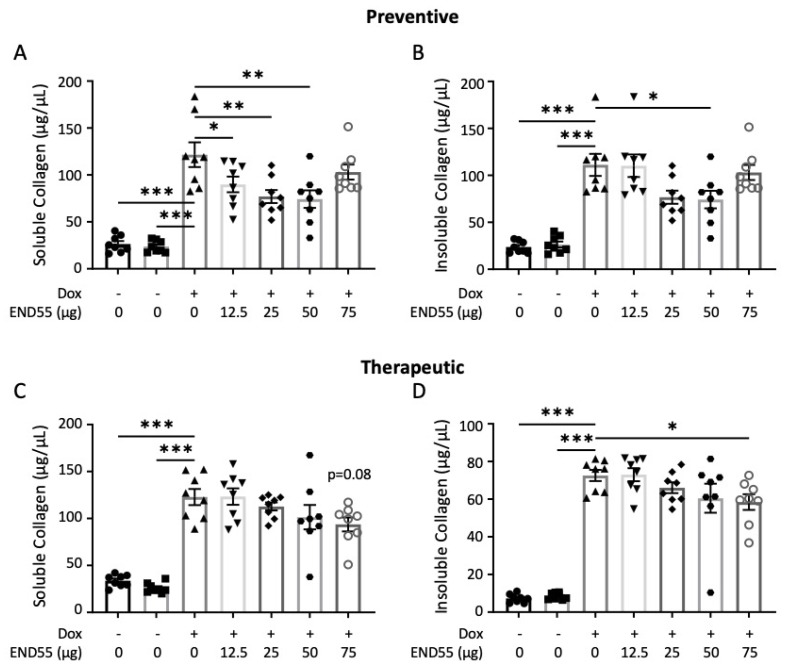
**Prophylactic and therapeutic effects of END55 in TGFβ1 transgenic mice.** Soluble (**A**,**C**) and insoluble (**B**,**D**) collagen was measured in tissues of mice receiving concurrent (prophylactic; (**A**,**B**)) and staggered (therapeutic; (**C**,**D**)) END55. N = 8. Data are represented as mean ± SD. * *p* < 0.05, ** *p* < 0.01, *** *p* < 0.001. Dox, doxycycline.

Next, we validated END55 in a second mouse model of pulmonary fibrosis involving doxycycline-inducible expression of bioactive human TGFβ1 in lung tissues [32]. TGFβ1 triple–transgenic mice received END55 (12.5–75 µg) via oral gavage biweekly for 21 days, either with doxycycline (prophylactic approach; Figure 5A,B) or starting 5 days after doxycycline (therapeutic approach; Figure 5C,D). In this model, we examined fibrosis via soluble and insoluble collagen content. Soluble collagen indicates the rate of newly synthesized material, whereas insoluble collagen indicates newly deposited material (comparable to hydroxyproline). Doxycycline administration significantly increased the amount of soluble and insoluble collagen in all experimental groups (Figure 5). Concurrent administration of doxycycline and END55 led to a significant reduction in both soluble collagen (12.5–50 μg END55, Figure 5A) and insoluble collagen (50 μg END55, Figure 5B), demonstrating the ability of END55 to reduce the fibrotic burden in these mice. In the therapeutic modality, END55 (75 μg) reduced soluble (Figure 5C) and insoluble (Figure 5D) collagen and reduced the expression of *Col1a1*, *Col1a2*, and Fn1 (Supplementary Figure S3), even when administered after the induction of TGFβ. Taken together, our data establish the therapeutic efficacy of END55 in a second murine model of pulmonary fibrosis.

### 2.7. END55 Reduces Markers of Fibrosis in Human Lung Tissue

**Figure 6:**
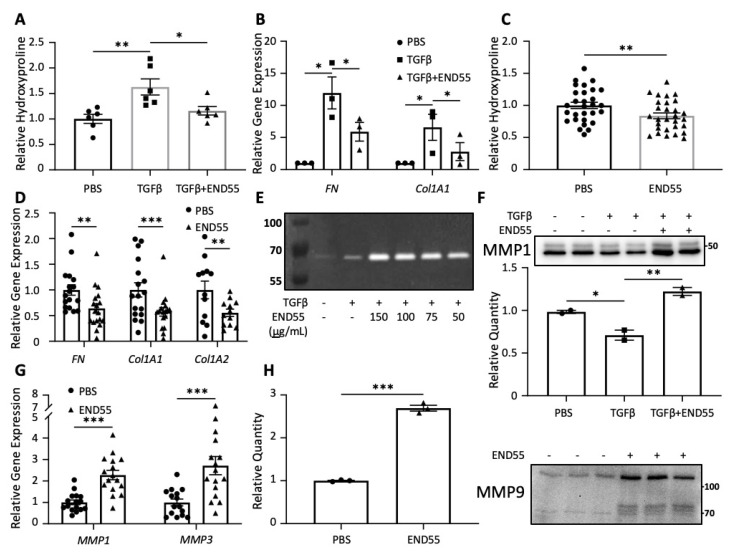
**END55 reduces fibrosis in human lung tissues and pulmonary fibroblasts.** (**A**) Hydroxyproline (HyP) in ex vivo normal lung tissues treated with TGFβ1 (10 ng/mL) ± END55 (122 µg/mL) for 72 h. N = 3. (**B**) *Fibronectin (FN*) and *Collagen* (*Col1A1*) gene expression normalized to *B2M* after treating normal lung fibroblasts with TGFβ1 ± END55 or PBS (vehicle) for 72 h. N = 3 donors. (**C**) HyP in IPF and SSc lung tissues treated with PBS (vehicle) or END55 (122 µg/mL) for 96 h. N = 15 donors. (**D**) Genes reduced by END55 (122 µg/mL) in IPF and SSc ex vivo organ culture after 48 h, normalized to housekeeping gene *GAPDH*. N = 6–9 donors. (**E**) Collagen zymography of 1 h supernatants from normal fibroblasts treated with TGFβ1 (10 ng/mL) ± END55 (concentrations indicated in µg/mL). N = 1. (**F**) MMP1 protein expression in normal lung tissue supernatants treated with PBS (vehicle), TGFβ1 (10 ng/mL), or TGFβ1+END55 (122 µg/mL) for 72 h. N = 2. (**G**) *MMP1* and *MMP3* gene expression (normalized to *GAPDH*) in IPF and SSc tissues treated with PBS (vehicle) or END55 (122 µg/mL) for 48 h. N = 8–9 donors. (**H**) MMP9 protein expression in ex vivo supernatants of SSc lung treated with PBS (vehicle) or END55 (122 µg/mL) for 144 h. N = 3. Data are represented as mean ± SD. * *p* < 0.05, ** *p* < 0.01, *** *p* < 0.001.

Normal and fibrotic lung tissues were used to test the efficacy of END55 in human organ models (Figure 6). Cores were generated from lung tissues and maintained in organ culture as we have previously described [30,33,34]. In the ex vivo organ model utilizing normal lung tissue, the pro-fibrotic agent TGFβ1 increased the hydroxyproline content of tissues maintained in organ culture for 72 h (Figure 6A). Concurrent treatment with END55 reduced the TGFβ1-mediated increase in hydroxyproline (Figure 6A), demonstrating that END55 can reduce TGFβ1-induced fibrosis in human lung tissues. Since an overabundance of ECM proteins, such as collagen and fibronectin, are a hallmark of fibrosis, the gene expression of these components was measured in lung fibroblasts outgrown from normal lung tissues (Figure 6B). In vitro, END55 abrogated the TGFβ1-mediated increase in *fibronectin* (*FN*) and *collagen 1A1* (*Col1A1*) gene expression in normal lung fibroblasts from different donors.

Since therapies tested in animal models often fail in human clinical trials, we sought to examine the effect of END55 on established human pulmonary fibrosis. Therefore, END55 was tested in fibrotic human lung cores derived from patients with IPF and SSc who underwent lung transplantation. These lungs represent severe end-stage disease requiring transplantation. END55 treatment of human fibrotic lung cores significantly reduced the hydroxyproline content of IPF and SSc lung tissues (Figure 6C), as well as the gene expression of *fibronectin*, *collagen 1A1*, and *collagen 1A2* (Figure 6D). These findings demonstrate that END55 can ameliorate fibrosis in human lung tissues from patients with end-stage fibrosis and is able to reverse established fibrosis in human tissues, thus providing direct relevance to the human disease.

### 2.8. END55 Increases Levels of Matrix Metalloproteases

Another aspect of the fibrotic process involves the remodeling of the ECM architecture, which is mediated in part by proteases, including MMPs [26]. MMP1, MMP3, and MMP9 have been reported to be elevated in the bronchoalveolar lavage fluid of IPF patients [35,36], and they contributed to fibrosis reversal in mouse livers [37]. MMP3 accelerated the degradation of cutaneous collagen in ex vivo murine skin models [38]. END55 was tested for its effect on select members of this family of proteases, including collagenases, gelatinases, and stromelysins [36].

We examined collagenases, specific mediators of the enzymatic cleavage of triple-helical collagen, using collagen zymography (Figure 6E). One-hour supernatants of normal lung fibroblasts treated with END55 fusion protein (50–150 µg/mL) showed a dose-dependent increase in band intensity at all END55 concentrations compared to vehicle or TGFβ1 alone (Figure 6E). Further, END55 increased secreted expression of MMP1, a typical collagenase, in the supernatants of lung tissue cores from normal human tissue in organ culture (Figure 6F).

Stromelysins are another type of MMP that can cleave proteins in the ECM. They differ from collagenases because they cannot cleave triple-helical collagens. The gene expression of both MMP1 (a collagenase) and MMP3 (a stromelysin) were significantly higher with END55 treatment after 48 h in ex vivo organ culture of IPF and SSc lung tissues (Figure 6G).

Gelatinases can cleave gelatin and some types of collagen. Secreted expression of MMP9, or gelatinase B, was significantly increased in the supernatants of ex vivo SSc lung tissues following a 144-h treatment with END55 (Figure 6H). Thus, END55 reduces hydroxyproline content and pro-fibrotic gene expression, and upregulates MMP-family proteases in normal and fibrotic human lung tissues, thereby reducing fibrosis and promoting ECM degradation.

### 2.9. END55 Decreases Fibrosis in Human Skin

**Figure 7:**
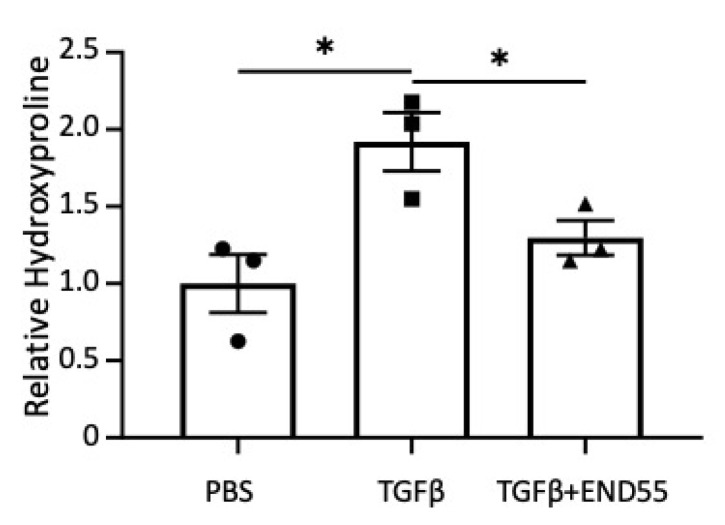
**END55 reduces fibrosis in human skin.** Hydroxyproline (HyP) levels in human skin maintained in organ culture and injected with PBS, TGFβ1 (10 ng/mL), or END55 (100 µg/mL) for 7 days. N = 3. Data are represented as mean ± SD. * *p* < 0.05.

To extend our findings with END55 to another human tissue, we tested the anti-fibrotic capacity of END55 in normal skin with fibrosis induced by TGFβ1, as previously described [20]. This model was optimized for testing the effects of pro- and anti-fibrotic factors in human tissue to show direct relevance to human disease. Human skin was injected with 10 ng/mL TGFβ1 and 100 µg END55. Skin punches were harvested from the injected area after 7 days. END55 reduced TGFβ1-stimulated hydroxyproline in normal skin in organ culture (Figure 7), demonstrating that END55 can ameliorate fibrosis in yet another human tissue.

## 3. Discussion

Fibrosis is a complication of several diseases that can affect nearly any organ and results in organ failure. We previously reported that endostatin and a peptide from its carboxyl-terminal region decrease skin thickness in TGFβ1-treated skin as well as in bleomycin mouse models [20]. We also reported that the peptide is orally available and activates the urokinase pathway [24,39]. The peptide was modified and manufactured in plants as an Fc-fusion protein. We show that the END55 fusion protein decreased fibrosis caused by bleomycin and TGFβ1 in two different mouse models. END55 also reduced established fibrosis in two separate human tissues: skin and lung. Additionally, END55 decreased gene expression of pro-fibrotic markers while stimulating the secretion of matrix metalloproteases known to break down ECM components. The efficacy of END55 in reducing fibrosis is likely due to its multi-pronged effect targeting several different key pathways in fibrosis [20,39].

Though circulating serum endostatin is increased in both IPF [40] and SSc [41,42,43], these levels are within the physiological range found in healthy people [44,45,46]. These findings suggest that endostatin levels in fibrosis cannot effectively reduce fibrosis, suggesting a “blunted” anti-fibrotic response. Full-length recombinant endostatin, with or without an amino-terminal nonamer, has been studied in the clinical setting for anti-angiogenic properties against various cancers, including gastric, nasopharyngeal, glioblastoma multiforme, lung–brain metastases, and two different lung cancers [47,48,49,50,51,52]. Endostatin has also ameliorated bleomycin-induced fibrosis in rats, CCl_4_-induced liver fibrosis in mice, hypertrophic scar formation in rabbit ears, and renal injury in streptozotocin diabetic rats [53,54,55,56,57]. We showed that endostatin also reduces TGFβ-induced fibrosis in human skin maintained in organ culture [22]. This study extends the functional properties of an endostatin fusion protein in the realm of anti-fibrosis therapeutics by testing the efficacy of a plant-manufactured fusion protein in response to different triggers of fibrosis.

From oxytocin to insulin, synthetic peptides have been used therapeutically for a variety of conditions. Peptides exhibit desirable attributes compared to small molecules based on their biological activity, specificity, membrane permeability, manufacturing cost, and enhanced delivery methods [58,59]. Over 60 therapeutic peptides have been approved for use, with hundreds more in clinical trials [59,60]. Peptides are being developed to treat a variety of human conditions, often for multiple implications, such as for neuroprotection [61,62,63,64,65], mitochondrial diseases [66,67], constipation [68], cancer [69,70,71,72,73], antimicrobials [74,75,76,77], and diabetes [78,79,80].

END55 was effective when administered orally. This effectiveness may be related to the large complexes that END55 forms (data not shown), which could protect against proteolytic degradation in the digestive tract. Alternatively, the fusion protein may be broken down to release a smaller active domain that may be more readily absorbed. Oral delivery of peptides is desirable and more practical than parenteral administration. Oral delivery is convenient, lacks discomfort, and has greater patient acceptance and adherence. Further, sterile conditions during manufacturing are not required for orally administered drugs, making oral formulations more cost-effective. Several approaches have been developed to facilitate oral delivery of peptides and proteins (reviewed in [81,82]). These approaches have supported successful oral delivery of several peptides/proteins, including semaglutide (a glucagon-like peptide 1 receptor agonist) for treating type 2 diabetes [78,79,81], desmopressin (an analog of vasopressin) for treating diabetes insipidus [60,81], and linaclotide for treating irritable bowel syndrome and constipation [60].

We describe the anti-fibrotic effects of an endostatin-derived fusion peptide produced in plants. Compared to the potentially difficult and costly prospect of manufacturing peptides by solid phase synthesis, recombinant expression in plants can provide a relatively inexpensive approach to producing high-quality material on a large scale. Although the manufacturing capacity and regulatory environment of recombinant protein production using microbial or animal cell expression systems are well established, they have several limitations, including impractically high costs or incomplete protein folding. In addition, animal cell systems require extensive lead time to develop production lines and rigorously clear potentially contaminating viruses. In contrast, transient expression systems in plants support rapid and inexpensive production of recombinant protein products. With the Launch approach we used to manufacture END55 in *Nicotiana benthamiana*, yields of purified protein can range from 200 to 1000 mg/kg of fresh plant biomass. A variety of recombinant protein targets, including molecules that require complex posttranslational modifications, have been produced in plants using this system. These targets include vaccine antigens, human biotherapeutic proteins, enzymes, monoclonal antibodies, and biologically active hormones. In addition, plants produce proteins and peptides that are appropriately folded without requiring the development of a refolding protocol. Such a protocol is often required for proteins expressed in bacteria and purified from inclusion bodies. Plant expression also produces the same range of co-translational and posttranslational modifications seen in other eukaryotic systems. In addition, plant expression of proteins and peptides is rapid and cost-effective. Further, a recombinant human glucocerebrosidase produced in carrot cells was approved by the FDA for enzyme replacement therapy of Gaucher’s disease in 2012. This approval confirmed the regulatory acceptance of using plant systems to create and produce biopharmaceuticals [17,18].

In summary, we have expanded our previous studies and identified a role for END55, a modified form of the E3 carboxyl-terminal region of endostatin, in modulating fibrosis. Overall, END55, when delivered by various routes, showed robust efficacy in ameliorating fibrosis in multiple murine models in which fibrosis was induced by different triggers. END55 also showed potency in human lung and skin tissues, reducing fibrosis and reversing ongoing mild and severe fibrosis. The initial mechanisms underlying END55 action include reducing pro-fibrotic gene expression and secretion of proteases that degrade ECM components. Thus, END55 is an attractive strategy for treating fibrosis due to its oral efficacy and its ability to ameliorate fibrosis in human tissues, thus providing direct relevance to human disease. Importantly, the use of endostatin peptides is attractive because the parent molecule endostatin, which has been used in clinical trials for cancer, has no toxicity and no drug resistance [83], and its production in plants offers a cost-effective strategy for the treatment of organ fibrosis.

## 4. Materials and Methods

### 4.1. Peptide

END55 peptide is an E3 Fc-fusion protein synthesized by Novici Biotech (Vacaville, CA, USA) using the Launch^TM^ transient plant expression system [84]. Briefly, the E3-Fc expression vector was transformed into *Agrobacterium tumefaciens* and then introduced via vacuum-infiltration into the leaves of *Nicotiana benthamiana*. After 5 days, infected tissues were harvested, homogenized in ice-cold buffer (50 mM Tris pH 8, 500 mM NaCl, 200 mM sucrose, 40 mM ascorbic acid, 5 mM EDTA, 2 mM PMSF), clarified by polypropylene/paper filter, and centrifuged at 20,000–30,000 rcf for 10 min. Protein was precipitated with sodium acetate (to pH 4.5) and neutralized with Tris pH 9, centrifuging after each step, and then sterile-filtered. E3-Fc was precipitated from the filtrate, mixed, cooled, centrifuged, and washed twice with 125 mM NaCl/1% PEG-8000. The pellet was solubilized and dialyzed in PBS with a Spectrum™ Spectra/Por™ Float-A Lyzer™ G2 (300 kDa cutoff), centrifuged, concentrated in a 10-kDa spin column (Millipore Amicon Ultra-15), and filter-sterilized. Purity was confirmed by SDS-PAGE. Purified E3-Fc is referred to as END55.

### 4.2. Animal Studies

Male mice aged 6–8 weeks were used for in vivo experiments following a protocol approved by the Institutional Animal Care and Use Committee of the Medical University of South Carolina or Yale University.

### 4.3. Bleomycin-Fibrosis Model

Bleomycin (Enzo Life Sciences, Teva Generics, or Hospira/Pfizer) was administered to C57BL/6J mice (The Jackson Laboratory) via the oropharyngeal route (1.2 mU/g body weight in ~50 µL) or by a dorsomedial osmotic pump for 7 days (0.33–15 mU/µL, 100 µL/week) as previously reported [85]. END55 was administered via oral gavage (30–60 µg/dose in ~50 µL), IV injection (100–500 µg/dose in 100 µL), intraperitoneal (IP) injection (100 µg/dose in 100 µL), or by dorsomedial osmotic pump for 7 days (100 µL/week).

TGFβ-fibrosis model: Expression of biologically active TGFβ in CC10-rtTA-tTS-TGF-β_1_ Mice was induced using doxycycline, as previously described [32]. Sex-matched mice on a C57BL/6 background were treated with 0–75 µg fusion protein (via oral gavage) biweekly for 21 days either with doxycycline to induce TGFβ1 production (prophylactic) or starting at day 5 following induction of TGFβ1 (therapeutic).

### 4.4. Collagen Assays

Collagen was measured using the hydroxyproline assay as previously described [24]. For murine tissues, the left lung was used to measure hydroxyproline. Sircol assay kits (BioColor Ltd., Carrickfergus, UK) were used per manufacturer instructions to assess the rates of newly synthesized (soluble) and deposited collagen (insoluble). Collagen concentrations were measured on a BioTek Synergy H1 plate reader or Bio-RAD SmartSpec^TM^ 3000.

### 4.5. Histology

Skin harvested pericentral to the injection or implant site of the mini-osmotic pump was fixed in 10% buffered formalin and embedded in paraffin. Sections (6 µM) of skin and lung tissues were stained with hematoxylin and eosin.

### 4.6. In Vitro and Ex Vivo Assays

Experiments involving skin and lungs from human donors were performed according to a protocol approved by the Institutional Review Board at the Medical University of South Carolina. Primary fibroblasts were isolated from normal organ donor lungs via the outgrowth method and maintained in Dulbecco’s Modified Eagle Medium (Corning Life Sciences, Corning, NY, USA), 10% fetal bovine serum (Sigma-Aldrich, Burlington, VT, USA), and 1% antibiotic/antimycotic (penicillin, streptomycin, and amphotericin B; ThermoFisher Scientific, Waltham, MA, USA) [7]. Lung tissues were cut into 5-mm diameter punches or cores and maintained in organ culture as previously described [34]. Skin tissues from normal donors undergoing abdominoplasty were maintained in organ culture in serum-free media as previously described [9,20], injected with treatments in a volume of 100 µL, cultured for 7 days, then harvested for analysis using 3-mm punches. Treatments were TGFβ1 (5–10 ng/mL; R&D Systems, Minneapolis, MN, USA) or vehicle (4 mM HCl + 0.1% bovine serum albumin; ThermoFisher Scientific, Waltham, MA, USA), and END55 or 1X PBS (Corning Life Sciences, Corning, NY, USA) as vehicle.

### 4.7. RNA Extraction and Real Time Polymerase Chain Reaction

Samples were homogenized with a BeadRuptor24 Homogenizer (Omni International, Kennesaw, GA, USA). RNA was extracted using an RNeasy Mini Kit (Qiagen, Germantown, TN, USA) per manufacturer instructions. Expression of genes was measured in cDNA prepared with the SuperScript™ IV first-strand synthesis system using TaqMan™ gene expression master mix (ThermoFisher Scientific, Waltham, MA, USA) and the following primers: *B2M* (Hs00187842_m1), *collagen 1A1* (Hs00164004_m1), *Col1A2* (Hs01028970_m1), *FN* (Hs00365052_m1), *GAPDH* (Hs02758991_g1), *MMP1* (Hs00899658_m1), and *MMP3* (Hs00968305_m1), mouse *Col1a1* (Mm00801666_g1), *Col1a2* (Mm00483888_m1), *Fn1* (Mm01256744_m1), *gapdh* (Mm99999915_g1), and *b2m* (Mm00437762_m1). PCR was performed on a StepOne Plus instrument (Applied Biosystems, Waltham, MA, USA). Data was analyzed using the delta–delta Ct method and normalized to the housekeeping gene *B2M* or *GAPDH*, as indicated.

### 4.8. Potency Assays

CTGF (Cloud Clone Corporation, Wuhan, PRC) and MMP1 (RayBiotech Life, Peachtree Corners, GA, USA) proteins were measured using enzyme-linked immunosorbent assay (ELISA) in supernatants of fetal lung fibroblasts (MRC5). LOX (Cloud Clone Corporation, Wuhan, China) was measured in mouse serum harvested 21 days after bleomycin treatment. Protein levels were measured on a BioTek Synergy H1 (BioTek, plate reader using commercially available sandwich ELISA kits per the manufacturer’s instructions. Supernatants were diluted 1:2 for the MMP1 assay. Concentrations were calculated from log-log standard curves generated with Gen5 2.09 software (Agilent, Santa Clara, CA, USA).

### 4.9. Zymography

Samples diluted in non-reducing sample buffer (1.0 M Tris, 80% glycerol, 0.6 g SDS, 1% bromophenol blue, pH 6.8) were resolved on 10% acrylamide gels containing 0.5 mg/mL rat tail collagen type I (Corning Life Sciences, Corning, NY, USA). Gels were washed twice with washing buffer (0.25% Triton X-100 in 50 mM Tris, pH 7.6), then once with incubation buffer (1% Triton X-100, 10 mM CaCl_2_, 0.2% sodium azide in 50 mM Tris, pH 7.6) for 15 min. Gels were then incubated overnight in fresh incubation buffer at 37 °C, stained with Coomassie blue (5% brilliant blue, 40% methanol, and 10% glacial acetic acid) at room temperature for 1 h, then destained (10% acetate, 40% methanol) with agitation and observed with transillumination.

### 4.10. Immunoblotting

Protein samples were run on SDS-PAGE and transferred to nitrocellulose membranes. Membranes were blocked with 5% non-fat dry milk for 1 h at room temperature, and then incubated with primary rabbit monoclonal anti-MMP1 (Ab134184, AbCam, Waltham, MA, USA) or mouse monoclonal anti-MMP9 (MAB911; R&D Systems, Minneapolis, MN, USA) antibody (1:1000) at 4 °C overnight. Membranes were then washed three times in TBS-Tween and incubated with HRP-conjugated anti-mouse (W4021; Cytiva, Marlborough, MA, USA) or anti-rabbit (NA934V, Cytiva, Marlborough, MA, USA) secondary antibody (1:5000–1:10,000) for 1 h at room temperature. Membranes were then washed three times and imaged on a FluorChemR Imager (ProteinSimple, Minneapolis, MN, USA) using Western Lightning™ ECL solution (PerkinElmer, Akron, OH, USA). Images were semi-quantified using ImageJ (NIH).

### 4.11. Statistical Analysis

Data were analyzed for statistical significance using a Student’s *t*-test test or one-way analysis of variance with an appropriate post-hoc test. Parametric tests were used since we did not observe large departures from normality in our data. A value of less than 0.05 was considered statistically significant, such that * *p* < 0.05, ** *p* < 0.01, *** *p* < 0.001, and **** *p* < 0.0001.

## Data Availability

Data is contained within the article and Supplementary Materials.

## Supplementary Materials

The following supporting information can be downloaded at: https://www.mdpi.com/article/10.3390/biomedicines10112861/s1. Figure S1: Intraperitoneal injections of END55 reduce Ashcroft scores in C57BL/6J mice treated with Bleomycin. Figure S2: Oral END55 reduces mRNA expression of fibrotic markers induced by bleomycin in C57BL/6J mouse lungs. Figure S3: END55 administered therapeutically reduces mRNA expression of fibrotic markers in TGFβ 1 transgenic mouse lungs.

## References

[1] Foundation PF https://www.pulmonaryfibrosis.org/.

[2] Association AL https://www.lung.org/.

[3] Wynn T.A. (2007). Common and unique mechanisms regulate fibrosis in various fibroproliferative diseases. J. Clin. Investig..

[4] Herzog E.L., Mathur A., Tager A.M., Feghali-Bostwick C., Schneider F., Varga J. (2014). Review: Interstitial lung disease associated with systemic sclerosis and idiopathic pulmonary fibrosis: How similar and distinct?. Arthritis Rheumatol..

[5] Raghu G., Chen S.-Y., Hou Q., Yeh W.-S., Collard H.R. (2016). Incidence and prevalence of idiopathic pulmonary fibrosis in US adults 18–64 years old. Eur. Respir. J..

[6] Perelas A., Silver R.M., Arrossi A.V., Highland K.B. (2020). Systemic sclerosis-associated interstitial lung disease. Lancet Respir. Med..

[7] Garrett S.M., Frost D.B., Feghali-Bostwick C. (2017). The mighty fibroblast and its utility in scleroderma research. J. Scleroderma Relat. Disord..

[8] Bonnans C., Chou J., Werb Z. (2014). Remodelling the extracellular matrix in development and disease. Nat. Rev. Mol. Cell Biol..

[9] Watanabe T., Frost D.B., Mlakar L., Heywood J., da Silveira W.A., Hardiman G., Feghali-Bostwick C. (2019). A Human Skin Model Recapitulates Systemic Sclerosis Dermal Fibrosis and Identifies COL22A1 as a TGFβ Early Response Gene that Mediates Fibroblast to Myofibroblast Transition. Genes.

[10] Denton C.P., Merkel P.A., Furst D.E., Khanna D., Emery P., Hsu V.M., Silliman N., Streisand J., Powell J., Åkesson A. (2006). Recombinant human anti–transforming growth factor β1 antibody therapy in systemic sclerosis: A multicenter, randomized, placebo-controlled phase I/II trial of CAT-192. Arthritis Care Res..

[11] Rice L.M., Padilla C.M., McLaughlin S.R., Mathes A.L., Ziemek J., Goummih S., Nakerakanti S., York M., Farina G., Whitfield M.L. (2015). Fresolimumab treatment decreases biomarkers and improves clinical symptoms in systemic sclerosis patients. J. Clin. Investig..

[12] King T.E., Bradford W.Z., Castro-Bernardini S., Fagan E.A., Glaspole I., Glassberg M.K., Gorina E., Hopkins P.M., Kardatzke D., Lancaster L. (2014). A phase 3 trial of pirfenidone in patients with idiopathic pulmonary fibrosis. N. Engl. J. Med..

[13] Richeldi L., Kolb M., Jouneau S., Wuyts W.A., Schinzel B., Stowasser S., Quaresma M., Raghu G. (2020). Efficacy and safety of nintedanib in patients with advanced idiopathic pulmonary fibrosis. BMC Pulm. Med..

[14] Richeldi L., Du Bois R.M., Raghu G., Azuma A., Brown K.K., Costabel U., Cottin V., Flaherty K.R., Hansell D.M., Inoue Y. (2014). Efficacy and safety of nintedanib in idiopathic pulmonary fibrosis. N. Engl. J. Med..

[15] Distler O., Highland K.B., Gahlemann M., Azuma A., Fischer A., Mayes M.D., Raghu G., Sauter W., Girard M., Alves M. (2019). Nintedanib for Systemic Sclerosis–Associated Interstitial Lung Disease. N. Engl. J. Med..

[16] Bathori G., Cervenak L., Karadi I. (2004). Caveolae—An alternative endocytotic pathway for targeted drug delivery. Crit. Rev. Ther. Drug Carr. Syst..

[17] Loh H.-S., Green B.J., Yusibov V. (2017). Using transgenic plants and modified plant viruses for the development of treatments for human diseases. Curr. Opin. Virol..

[18] Yao J., Weng Y., Dickey A., Wang K.Y. (2015). Plants as Factories for Human Pharmaceuticals: Applications and Challenges. Int. J. Mol. Sci..

[19] Ren H.T., Li Y., Wang S.D., Han C.M. (2017). Effects of endostatin pretreatment on fibrosis of human skin fibroblasts and the mechanisms. Zhonghua Shao Shang Za Zhi..

[20] Yamaguchi Y., Takihara T., Chambers R.A., Veraldi K.L., Larregina A.T., Feghali-Bostwick C.A. (2012). A Peptide Derived from Endostatin Ameliorates Organ Fibrosis. Sci. Transl. Med..

[21] O’Reilly M.S., Boehm T., Shing Y., Fukai N., Vasios G., Lane W.S., Flynn E., Birkhead J.R., Olsen B.R., Folkman J. (1997). Endostatin: An endogenous inhibitor of angiogenesis and tumor growth. Cell.

[22] Chang J.-H., Javier J.A., Chang G.-Y., Oliveira H.B., Azar D.T. (2005). Functional characterization of neostatins, the MMP-derived, enzymatic cleavage products of type XVIII collagen. FEBS Lett..

[23] Ichinose K., Maeshima Y., Yamamoto Y., Kitayama H., Takazawa Y., Hirokoshi K., Sugiyama H., Yamasaki Y., Eguchi K., Makino H. (2005). Antiangiogenic endostatin peptide ameliorates renal alterations in the early stage of a type 1 diabetic nephropathy model. Diabetes.

[24] Nishimoto T., Mlakar L., Takihara T., Feghali-Bostwick C. (2015). An endostatin-derived peptide orally exerts anti-fibrotic activity in a murine pulmonary fibrosis model. Int. Immunopharmacol..

[25] Assassi S., Radstake T.R., Mayes M.D., Martin J. (2013). Genetics of scleroderma: Implications for personalized medicine?. BMC Med..

[26] Theocharis A.D., Skandalis S.S., Gialeli C., Karamanos N.K. (2016). Extracellular matrix structure. Adv. Drug Deliv. Rev..

[27] Tjin G., White E.S., Faiz A., Sicard D., Tschumperlin D.J., Mahar A., Kable E.P.W., Burgess J.K. (2017). Lysyl oxidases regulate fibrillar collagen remodelling in idiopathic pulmonary fibrosis. Dis. Model. Mech..

[28] Philp C.J., Siebeke I., Clements D., Miller S., Habgood A., John A.E., Navaratnam V., Hubbard R.B., Jenkins G., Johnson S.R. (2018). Extracellular Matrix Cross-Linking Enhances Fibroblast Growth and Protects against Matrix Proteolysis in Lung Fibrosis. Am. J. Respir. Cell Mol. Biol..

[29] Rimar D., Rosner I., Nov Y., Slobodin G., Rozenbaum M., Halasz K., Haj T., Jiries N., Kaly L., Boulman N. (2013). Brief report: Lysyl oxidase is a potential biomarker of fibrosis in systemic sclerosis. Arthritis Rheumatol..

[30] Nguyen X.-X.M., Nishimoto T., Takihara T., Mlakar L., Bradshaw A.D., Feghali-Bostwick C.A. (2021). Lysyl oxidase directly contributes to extracellular matrix production and fibrosis in systemic sclerosis. Am. J. Physiol. Cell Mol. Physiol..

[31] Ashcroft T., Simpson J.M., Timbrell V. (1988). Simple method of estimating severity of pulmonary fibrosis on a numerical scale. J. Clin. Pathol..

[32] Lee C.G., Cho S.J., Kang M.J., Chapoval S.P., Lee P.J., Noble P.W., Yehualaeshet T., Lu B., Flavell R.A., Milbrandt J. (2004). Early growth response gene 1–mediated apoptosis is essential for transforming growth factor β1–induced pulmonary fibrosis. J. Exp. Med..

[33] Su Y., Nishimoto T., Hoffman S., Nguyen X.-X., Pilewski J.M., Feghali-Bostwick C. (2019). Insulin-like growth factor binding protein-4 exerts antifibrotic activity by reducing levels of connective tissue growth factor and the C-X-C chemokine receptor 4. FASEB BioAdv..

[34] Nguyen X.-X., Muhammad L., Nietert P.J., Feghali-Bostwick C. (2018). IGFBP-5 Promotes Fibrosis via Increasing Its Own Expression and That of Other Pro-fibrotic Mediators. Front. Endocrinol..

[35] Morais A., Beltrão M., Sokhatska O., Costa D., Melo N., Mota P., Marques A., Delgado L. (2015). Serum metalloproteinases 1 and 7 in the diagnosis of idiopathic pulmonary fibrosis and other interstitial pneumonias. Respir. Med..

[36] Mahalanobish S., Saha S., Dutta S., Sil P.C., Mahalanobish S., Saha S., Dutta S., Sil P.C. (2020). Matrix metalloproteinase: An upcoming therapeutic approach for idiopathic pulmonary fibrosis. Pharmacol. Res..

[37] Popov Y.V., Sverdlov D.Y., Bhaskar K.R., Sharma A.K., Millonig G., Patsenker E., Krahenbuhl S., Krahenbuhl L., Schuppan D. (2010). Macrophage-mediated phagocytosis of apoptotic cholangiocytes contributes to reversal of experimental biliary fibrosis. Am. J. Physiol. Liver Physiol..

[38] Mirastschijski U., Lupše B., Maedler K., Sarma B., Radtke A., Belge G., Dorsch M., Wedekind D., McCawley L.J., Boehm G. (2019). Matrix Metalloproteinase-3 is Key Effector of TNF-α-Induced Collagen Degradation in Skin. Int. J. Mol. Sci..

[39] Sharma S., Watanabe T., Nishimoto T., Takihara T., Mlakar L., Nguyen X.-X., Sanderson M., Su Y., Chambers R.A., Feghali-Bostwick C. (2021). E4 engages uPAR and enolase-1 and activates urokinase to exert antifibrotic effects. JCI Insight.

[40] Sumi M., Satoh H., Kagohashi K., Ishikawa H., Sekizawa K. (2005). Increased serum levels of endostatin in patients with idiopathic pulmonary fibrosis. J. Clin. Lab. Anal..

[41] Hummers L.K., Hall A., Wigley F.M., Simons M. (2009). Abnormalities in the regulators of angiogenesis in patients with scleroderma. J. Rheumatol..

[42] Reiseter S., Molberg Ø., Gunnarsson R., Lund M.B., Aalokken T.M., Aukrust P., Ueland T., Garen T., Brunborg C., Michelsen A. (2015). Associations between circulating endostatin levels and vascular organ damage in systemic sclerosis and mixed connective tissue disease: An observational study. Arthritis Res. Ther..

[43] Dziankowska-Bartkowiak B., Waszczykowska E., Zalewska A., Sysa-Jędrzejowska A. (2005). Correlation of endostatin and tissue inhibitor of metalloproteinases 2 (TIMP2) serum levels with cardiovascular involvement in systemic sclerosis patients. Mediat. Inflamm..

[44] Feldman A., Tamarkin L., Paciotti G.F., Simpson B.W., Linehan W.M., Yang J.C., Fogler W.E., Turner E.M., Alexander H.R., Libutti S.K. (2000). Serum endostatin levels are elevated and correlate with serum vascular endothelial growth factor levels in patients with stage IV clear cell renal cancer. Clin. Cancer Res..

[45] Bono P., Teerenhovi L., Joensuu H. (2003). Elevated serum endostatin is associated with poor outcome in patients with non-Hodgkin lymphoma. Cancer.

[46] Feldman A., Pak H., Yang J.C., Alexander H.R., Libutti S.K. (2001). Serum endostatin levels are elevated in patients with soft tissue sarcoma. Cancer.

[47] Yao J., Fan L., Peng C., Huang A., Liu T., Lin Z., Yang Q., Zhang T., Ma H. (2017). Clinical efficacy of endostar combined with chemotherapy in the treatment of peritoneal carcinomatosis in gastric cancer: Results from a retrospective study. Oncotarget.

[48] Xu H., Lv D., Meng Y., Wang M., Wang W., Zhou C., Zhou S., Chen X., Yang H. (2020). Endostar improved efficacy of concurrent chemoradiotherapy with vinorelbine plus carboplatin in locally advanced lung squamous cell carcinoma patients with high serum Lp(a) concentration. Ann. Palliat. Med..

[49] Honglian M., Zhouguang H., Fang P., Lujun Z., Dongming L., Yujin X., Yong B., Liming X., YiRui Z., Xiao H. (2020). Different administration routes of recombinant human endostatin combined with concurrent chemoradiotherapy might lead to different efficacy and safety profile in unresectable stage III non-small cell lung cancer: Updated follow-up results from two phase II trials. Thorac. Cancer.

[50] Li Y., Tian Y., Jin F., Wu W., Long J., Ouyang J., Zhou Y. (2019). A phase II multicenter randomized controlled trial to compare standard chemoradiation with or without recombinant human endostatin injection (Endostar) therapy for the treatment of locally advanced nasopharyngeal carcinoma: Long-term outcomes update. Curr. Probl. Cancer.

[51] Szentirmai O., Baker C.H., Bullain S.S., Lin N., Takahashi M., Folkman J., Mulligan R.C., Carter B.S. (2008). Successful inhibition of intracranial human glioblastoma multiforme xenograft growth via systemic adenoviral delivery of soluble endostatin and soluble vascular endothelial growth factor receptor-2. J. Neurosurg..

[52] Jiang X., Ding M., Qiao Y., Liu Y., Liu L. (2013). Recombinant human endostatin combined with radiotherapy in the treatment of brain metastases of non-small cell lung cancer. Clin. Transl. Oncol..

[53] Wan Y.-Y., Tian G.-Y., Guo H.-S., Kang Y.-M., Yao Z.-H., Li X.-L., Liu Q.-H., Lin D.-J. (2013). Endostatin, an angiogenesis inhibitor, ameliorates bleomycin-induced pulmonary fibrosis in rats. Respir. Res..

[54] Chen J., Liu D.-G., Yang G., Kong L.-J., Du Y.-J., Wang H.-Y., Li F.-D., Pei F.-H., Song J.-T., Fan Y.-J. (2014). Endostar, a novel human recombinant endostatin, attenuates liver fibrosis in CCl_4_-induced mice. Exp. Biol. Med..

[55] Zhiyong W., Fei S., Lianju X., Yingkai L., Chun Q., Shuliang L., Xiqiao W. (2012). Endostar injection inhibits rabbit ear hypertrophic scar formation. Int. J. Low. Extrem. Wounds.

[56] Ren H.-T., Hu H., Li Y., Jiang H.-F., Hu X.-L., Han C.-M. (2013). Endostatin inhibits hypertrophic scarring in a rabbit ear model. J. Zhejiang Univ. Sci. B.

[57] Bai X., Li X., Tian J., Zhou Z. (2014). Antiangiogenic treatment diminishes renal injury and dysfunction via regulation of local AKT in early experimental diabetes. PLoS ONE.

[58] Baig M.H., Ahmad K., Saeed M., Alharbi A.M., Barreto G.E., Ashraf G.M., Choi I. (2018). Peptide based therapeutics and their use for the treatment of neurodegenerative and other diseases. Biomed. Pharmacother..

[59] Danho W., Swistok J., Khan W., Chu X.-J., Cheung A., Fry D., Sun H., Kurylko G., Rumennik L., Cefalu J. (2009). Opportunities and challenges of developing peptide drugs in the pharmaceutical industry. Adv. Exp. Med. Biol..

[60] Lau J.L., Dunn M.K. (2018). Therapeutic peptides: Historical perspectives, current development trends, and future directions. Bioorg. Med. Chem..

[61] Knight P.D., Karamanos T., Radford S., Ashcroft A.E. (2017). Identification of a novel site of interaction between ataxin-3 and the amyloid aggregation inhibitor polyglutamine binding peptide 1. Eur. J. Mass Spectrom..

[62] Sragovich S., Amram N., Yeheskel A., Gozes I. (2020). VIP/PACAP-Based Drug Development: The ADNP/NAP-Derived Mirror Peptides SKIP and D-SKIP Exhibit Distinctive in vivo and in silico Effects. Front. Cell Neurosci..

[63] Igarashi H., Ito T., Mantey S.A., Pradhan T.K., Hou W., Coy D.H., Jensen R.T. (2005). Development of simplified vasoactive intestinal peptide analogs with receptor selectivity and stability for human vasoactive intestinal peptide/pituitary adenylate Cyclase-activating polypeptide receptors. J. Pharmacol. Exp. Ther..

[64] Martins N., Ferreira D., Rodrigues M.C., Cintra A., Santos N., Sampaio S., Santos A. (2010). Low-molecular-mass peptides from the venom of the Amazonian viper Bothrops atrox protect against brain mitochondrial swelling in rat: Potential for neuroprotection. Toxicon.

[65] Morimoto B.H., Fox A.W., Stewart A.J., Gold M. (2013). Davunetide: A review of safety and efficacy data with a focus on neurodegenerative diseases. Expert Rev. Clin. Pharmacol..

[66] Disatnik M., Ferreira J.C., Campos J.C., Gomes K.S., Dourado P.M., Qi X., Mochly-Rosen D. (2013). Acute inhibition of excessive mitochondrial fission after myocardial infarction prevents long-term cardiac dysfunction. J. Am. Heart Assoc..

[67] Filichia E., Hoffer B., Qi X., Luo Y. (2016). Inhibition of Drp1 mitochondrial translocation provides neural protection in dopaminergic system in a Parkinson’s disease model induced by MPTP. Sci. Rep..

[68] Barish C., Dorn S., Fogel R.P., Patel R., Rosenberg J. (2020). Plecanatide Is Effective and Safe in the Treatment for Chronic Idiopathic Constipation: Results of a Phase II Trial. Am. J. Dig. Dis..

[69] Engel J.B., Tinneberg H.-R., Rick F.G., Berkes E., Schally A.V. (2016). Targeting of Peptide Cytotoxins to LHRH Receptors for Treatment of Cancer. Curr. Drug Targets.

[70] Enokida H., Yamada Y., Tatarano S., Yoshino H., Yonemori M., Sakaguchi T., Nishimura H., Eura R., Nakagawa M. (2019). Oncological outcome of neoadjuvant low-dose estramustine plus LHRH agonist/antagonist followed by extended radical prostatectomy for Japanese patients with high-risk localized prostate cancer: A prospective single-arm study. Jpn. J. Clin. Oncol..

[71] Schally A.V., Wang H., He J., Cai R., Sha W., Popovics P., Perez R., Vidaurre I., Zhang X. (2018). Agonists of growth hormone-releasing hormone (GHRH) inhibit human experimental cancers in vivo by down-regulating receptors for GHRH. Proc. Natl. Acad. Sci. USA.

[72] Ren S.X., Cheng A.S., To K.F., Tong J.H., Li M.S., Shen J., Wong C.C., Zhang L., Chan R.L., Wang X.J. (2012). Host immune defense peptide LL-37 activates caspase-independent apoptosis and suppresses colon cancer. Cancer Res..

[73] Valentinis B., Porcellini S., Asperti C., Cota M., Zhou D., Di Matteo P., Garau G., Zucchelli C., Avanzi N.R., Rizzardi G.P. (2019). Mechanism of Action of the Tumor Vessel Targeting Agent NGR-hTNF: Role of Both NGR Peptide and hTNF in Cell Binding and Signaling. Int. J. Mol. Sci..

[74] Zhang L., Wu W.K.K., Gallo R.L., Fang E.F., Hu W., Ling T.K.W., Shen J., Chan R.L.Y., Lu L., Luo X.M. (2016). Critical Role of Antimicrobial Peptide Cathelicidin for Controlling *Helicobacter pylori* Survival and Infection. J. Immunol..

[75] Wei H.-M., Lin L.-C., Wang C.-F., Lee Y.-J., Chen Y.-T., Liao Y.-D. (2016). Antimicrobial Properties of an Immunomodulator—15 kDa Human Granulysin. PLoS ONE.

[76] Akram A.R., Avlonitis N., Scholefield E., Vendrell M., McDonald N., Aslam T., Craven T.H., Gray C., Collie D.S., Fisher A.J. (2019). Enhanced avidity from a multivalent fluorescent antimicrobial peptide enables pathogen detection in a human lung model. Sci. Rep..

[77] Nilsson A.C., Janson H., Wold H., Fugelli A., Andersson K., Håkangård C., Olsson P., Olsen W.M. (2015). LTX-109 is a novel agent for nasal decolonization of methicillin-resistant and -sensitive *Staphylococcus aureus*. Antimicrob. Agents Chemother..

[78] Manandhar B., Ahn J.-M. (2014). Glucagon-like peptide-1 (GLP-1) analogs: Recent advances, new possibilities, and therapeutic implications. J. Med. Chem..

[79] Morieri M.L., Frison V., Rigato M., D’Ambrosio M., Tadiotto F., Paccagnella A., Simioni N., Lapolla A., Avogaro A., Fadini G.P. (2020). Effectiveness of Dulaglutide in the Real World and in Special Populations of Type 2 Diabetic Patients. J. Clin. Endocrinol. Metab..

[80] Pozzilli P., Bosi E., Cirkel D., Harris J., Leech N., Tinahones F.J., Vantyghem M.-C., Vlasakakis G., Ziegler A.-G., Janmohamed S. (2020). Randomized 52-week Phase 2 Trial of Albiglutide Versus Placebo in Adult Patients with Newly Diagnosed Type 1 Diabetes. J. Clin. Endocrinol. Metab..

[81] Drucker D.J. (2019). Advances in oral peptide therapeutics. Nat. Rev. Drug Discov..

[82] Shaji J., Patole V. (2008). Protein and peptide drug delivery: Oral approaches. Indian J. Pharm. Sci..

[83] Boehm T., Folkman J., Browder T., O’Reilly M.S. (1997). Antiangiogenic therapy of experimental cancer does not induce acquired drug resistance. Nature.

[84] Musiychuk K., Stephenson N., Bi H., Farrance C.E., Orozovic G., Brodelius M., Brodelius P., Horsey A., Ugulava N., Shamloul A. (2007). A launch vector for the production of vaccine antigens in plants. Influenza Other Respir. Viruses.

[85] Watanabe T., Nishimoto T., Mlakar L., Heywood J., Malaab M., Hoffman S., Feghali-Bostwick C. (2017). Optimization of a murine and human tissue model to recapitulate dermal and pulmonary features of systemic sclerosis. PLoS ONE.

