# Supplementary material for: Ameliorating Fibrosis in Murine and Human Tissues with END55, an Endostatin-Derived Fusion Protein Made in Plants
Source: Biomedicines. 2022 Nov 9;10(11):2861. doi: 10.3390/biomedicines10112861 (PMC9687961; doi:10.3390/biomedicines10112861)
Supplement: Supplementary file 1 [file biomedicines-10-02861-s001.zip › biomedicines-1922520-supplementary.pdf]

SUPPLEMENTARY INFORMATION

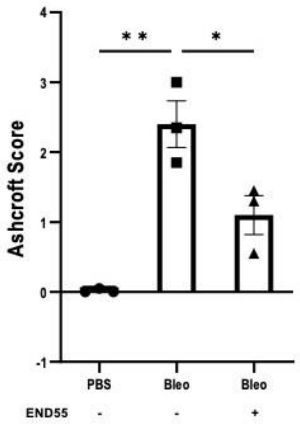

Supplementary Figure S1: **Intraperitoneal injections of END55 reduce Ashcroft scores in C57BL/6J mice treated with Bleomycin**. Bleomycin was administered in PBS via oropharyngeal route at 1.2 mU/g of mouse bodyweight on day zero. END55 was administered in PBS via intraperitoneal injections at 100 µg/100 µL on days four, seven, ten, and fourteen. Mice were euthanized on day seventeen. Right lung lobes were fixed, paraffin embedded, and sectioned. Sections were stained with H&E. N = 3 per treatment group. Data are represented as mean ± SE. *p < 0.05, **p < 0.005.

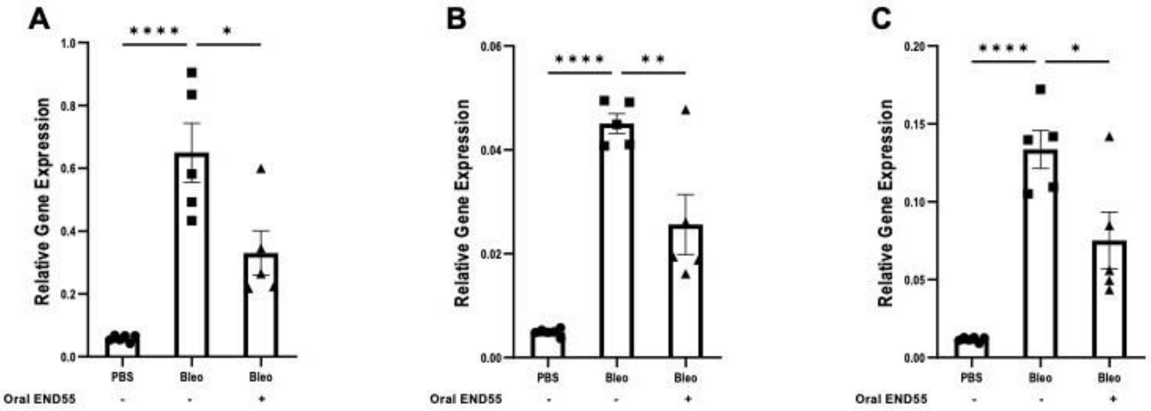

Supplemental Figure S2. Oral END55 reduces mRNA expression of fibrotic markers induced by bleomycin in C57BL/6J mouse lungs. Bleomycin was administered in PBS via oropharyngeal route at 1.0 mU/g of mouse bodyweight on day zero. END55 was administered in PBS via oral gavage at 50 µg/100 µL every 72 hr from day zero to day twelve. Mice were euthanized on day fourteen. RNA was extracted from left lungs and analyzed via qPCR. (**A**) *Col1a1* mRNA levels; (**B**) *Col1a2* mRNA levels; (**C**) *Fn1* mRNA levels, all relative to the expression of *gapdh*. N = 5-6 per treatment group. Data are represented as mean ± SE. *p < 0.05, **p < 0.005, ****p < 0.0001.

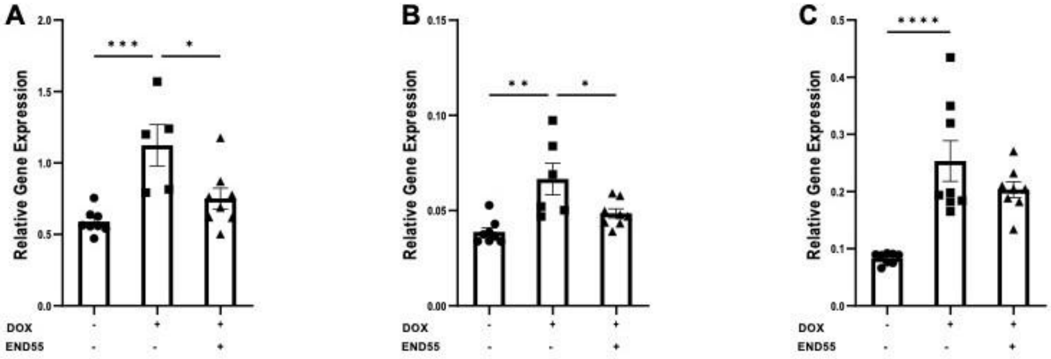

Supplemental Figure S3. END55 administered therapeutically reduces mRNA expression of fibrotic markers in TGFβ1 transgenic mouse lungs. TGFβ1 expression was induced by administration of doxycycline (DOX), and END55 (75 µg/ dose) was administered via oral gavage after 5 days. RNA was extracted from lung tissues and analyzed by qPCR(**A**) *Col1a1* mRNA levels; (**B**) *Col1a2* mRNA levels; (**C**) *Fn1* mRNA levels, all relative to the expression of *b2m*. N = 5-8 per treatment group. Data are represented as mean ± SE. *p < 0.05, **p < 0.005, ***p<0.0005, ****p < 0.0001.
